# Segatella asaccharophila sp. nov., an anaerobic pectinophile isolated from a two-phase methane fermentation system

**DOI:** 10.1099/ijsem.0.006606

**Published:** 2024-12-18

**Authors:** Tomoki Makiura, Hou-Chia Tseng, Naoshi Fujimoto, Akihiro Ohnishi

**Affiliations:** 1 Department of Fermentation Science, Faculty of Applied Bioscience, Tokyo University of Agriculture, 1-1-1 Sakuragaoka, Setagaya, Tokyo, 156-8502, Japan

**Keywords:** asaccharolytic, pectin, pectinophile, *Segatella*, succinate

## Abstract

An obligately anaerobic, Gram-stain-negative, non-spore-forming and non-motile rod (strain LPYR103-Pre^T^) was isolated from a two-phase methane fermentation system. Using 16S rRNA gene sequence-based phylogenetic analysis, strain LPYR103-Pre^T^ was classified in the genus *Segatella*. The 16S rRNA gene sequence similarity, average nucleotide identity and digital DNA–DNA hybridization between strain LPYR103-Pre^T^ and its phylogenetically nearest species *– Segatella cerevisiae* JCM 30867^T^ – were 94.4, 77.9 and 23.4%, respectively. The genome size of strain LPYR103-Pre^T^ was 3 326 733 bp, and its genomic DNA G+C content was 44.05%. The most abundant cellular fatty acid was anteiso-C_15 : 0_. The growth of strain LPYR103-Pre^T^ was stimulated by the addition of pectin, d-galacturonate and d-glucuronate; in contrast, the strain exhibited poor growth in the presence of common sugars, such as glucose. Therefore, strain LPYR103-Pre^T^ was classified as a pectinophile – a bacterium that shows a preference for pectin and a few related compounds as substrates. Glucose is degraded by type strains of 12 species belonging to the genus *Segatella*; thus, strain LPYR103-Pre^T^ is the first described pectinophile belonging to this genus. Strain LPYR103-Pre^T^ produced succinate and acetate as its major metabolic end products. Based on the differences in the phylogenetic, genomic, physiological and chemotaxonomic characteristics of strain LPYR103-Pre^T^ and related species, the name *Segatella asaccharophila* sp. nov. is proposed to accommodate strain LPYR103-Pre^T^ (= NRIC 0997^T^ = JCM 37351^T^=DSM 118531^T^ = KCTC 25923^T^).

## Introduction

Anaerobic digestion is a biological treatment process that converts organic waste and wastewater into biogas, mainly methane and carbon dioxide, in the absence of oxygen. Previously, we reported a small-scale, two-phase methane fermentation system equipped with solubilization treatment (throughput: 100 kg garbage per day) [1]. This system consisted of four units. The garbage (food waste) fed into the system was first solubilized in an aero-solubilization tank (Unit 1). Next, acidogenic fermentation was performed in an acidification tank (Unit 2), followed by the precipitation of undecomposed and insoluble substances in a gravity sedimentation tank (Unit 3), which was then supplied to an expanded granular sludge bed reactor (Unit 4). We analysed the levels of short-chain fatty acids (SCFAs) and microbiota in each unit of this system. The main SCFAs found in the acidification tank were lactate, acetate and butyrate. The bacterial microbiota was mainly composed of 17 genera, including lactic acid bacteria (genera *Lactobacillus* and *Bifidobacterium*), butyric acid bacteria (genus *Clostridium*), lactate-utilizing bacteria (genera *Veillonella* and *Megasphaera*) and an uncharacterized *Segatella* sp. This complex microbial community was enriched in a pectin-containing medium, and *Segatella* sp. LPYR103-Pre^T^ (= NRIC 0997^T^ = JCM 37351^T^=DSM 118531^T^ = KCTC 25923^T^) was isolated [2].

The genus *Segatella* is a newly proposed genus, defined following the reclassification of species in the genus *Prevotella*. Certain species previously assigned to the genus *Prevotella* have now been reclassified into the genus *Segatella* [3]. As of May 2024, the genus *Segatella* contains 12 species that can ferment a wide range of common sugars, such as glucose and lactose [4,13]. However, strain LPYR103-Pre^T^ showed poor growth in the presence of common sugars. Interestingly, the growth of strain LPYR103-Pre^T^ was stimulated in the presence of pectin, d-galacturonate or d-glucuronate [2], indicating that it is a pectinophile. *Lachnospira pectinoschiza*, *Bacteroides pectinophilus*, *Bacteriodes galacturonicus*, *Treponema pectinovorum*, *Eubacterium* sp. strain P-1, *Monoglobus pectinilyticus*, *Natranaerovirga pectinivora* and *Natranaerovirga hydrolytica* are pectinophiles, exhibiting a preference for pectin and a limited range of related compounds [14,21]. To the best of our knowledge, strain LPYR103-Pre^T^ is the first pectinophile belonging to the genus *Segatella*. Here, we report the phylogenetic, genomic, physiological and chemotaxonomic characteristics of strain LPYR103-Pre^T^.

## Methods

### Isolation, maintenance and culture conditions

**Fig. 1.**
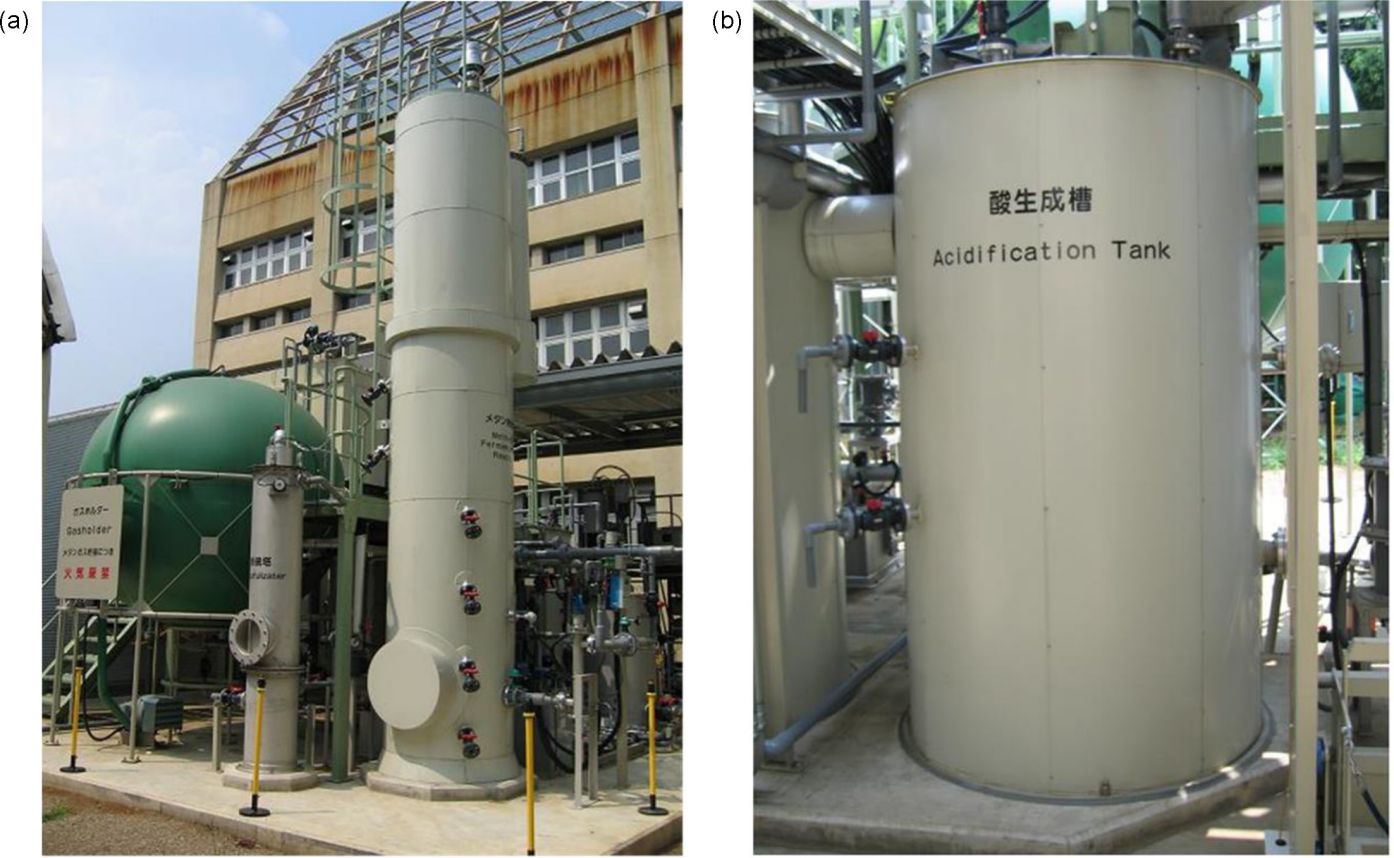
Two-phase methane fermentation system equipped with solubilization treatment at the Tokyo University of Agriculture. (**a**) Overview. (**b**) Acidification tank.

Strain LPYR103-Pre^T^ was isolated from anaerobic digestion sludge obtained from an acidification tank (35°38′31″N, 139°37′48″E) within a two-phase methane fermentation system equipped with solubilization treatment, at the Tokyo University of Agriculture, Japan (Fig. 1). Strains LPYR103-Pre^T^ and *Segatella cerevisiae* JCM 30867^T^ used in this study were maintained on lactate–yeast extract–peptone–pectin (LYPP) agar plates [2] under anaerobic conditions at 37 °C for 7 days, using an AnaeroPack system (Mitsubishi Gas Chemical Company, Inc., Tokyo, Japan).

### 16S rRNA gene sequencing and phylogenetic analysis

The genomic DNA of strain LPYR103-Pre^T^ was extracted using a DNeasy PowerSoil Kit (QIAGEN GmbH, Hilden, Germany) and used for 16S rRNA gene sequencing. The 16S rRNA gene was amplified using the primer pair 20F (5′-AGTTTGATCATGGCTCA-3′) and 1540R (5′-AAGGAGGTGATCCAACCGCA-3′). PCR amplification was performed as previously described [22], with minor modifications. The following PCR amplification conditions were used: initial heat polymerase activation at 95 °C for 3 min, followed by 30 cycles at 95 °C for 30 s (denaturation), 55 °C for 30 s (annealing), 72 °C for 1 min (extension) and a final extension at 72 °C for 5 min. The PCR products were stored at 4 °C until further use. Sequencing was performed using the BigDye Terminator version 1.1 Cycle Sequencing Kit (Thermo Fisher Scientific, Waltham, MA, USA) on an ABI PRISM Model 310 Genetic Analyzer (Applied Biosystems, Foster City, CA, USA). The obtained nearly complete 16S rRNA gene sequence (1484 bp) was subjected to blast in the DDBJ/ENA/GenBank database and EzBioCloud server (https://www.ezbiocloud.net/) [23,24]. Multiple sequence alignments were performed using ClustalX (version 2.1) [25]. Phylogenetic distances (*K*_nuc_) between the aligned sequences were calculated using Kimura’s two-parameter method [26]. The neighbour-joining method [27] was used to construct a phylogenetic tree. The topology of the phylogenetic tree was evaluated by bootstrapping with 1000 replications [28].

### Genomic analysis

Genomic analysis of the strain LPYR103-Pre^T^ was performed according to a previously described method [2]. According to the proposed minimum standards for taxonomic purposes [29], average nucleotide identity (ANI) and digital DNA–DNA hybridization (dDDH) values were calculated using the ANI calculator (www.ezbiocloud.net/tools/ani) [30] and formula 2 (identities/HSP length) of the Genome-to-Genome Distance Calculator 3.0 (https://ggdc.dsmz.de/ggdc.php) [31], respectively. The Type (Strain) Genome Server (TYGS) of the Leibniz Institute DSMZ was used for whole-genome sequence-based phylogenomic analysis [32,33].

### Physiological and chemotaxonomic analysis

The cellular, physiological and chemotaxonomic characteristics of strain LPYR103-Pre^T^ were examined. Oxygen tolerance was determined by aerobic cultivation using pectin agar plates [2]. Spore formation was assessed by measuring the growth of the cells exposed to 80 °C for 10 min [12]. Cell motility was observed using a phase-contrast microscope. The effects of incubation temperature, initial pH and NaCl concentrations on the growth potential of strain LPYR103-Pre^T^ were evaluated using batch tests [2]. The optimum temperature for the growth was determined using LYPP medium [2] at different temperatures (25, 30, 34, 35, 36, 37, 40 and 45 °C). The optimum pH for the growth was determined using LYPP medium at 37 °C, with variable pH values of 3.03, 3.53, 4.02, 4.50, 4.77, 5.04, 5.24, 5.51, 5.68, 5.85, 5.96, 6.05, 6.39, 6.92, 7.09 and 7.68 (values verified after autoclaving). The effect of NaCl on growth was determined using variable NaCl concentrations (0.0–3.0 %, w/v) in LYPP medium without sodium lactate solution. Physiological reactions were determined using API 20 A, API ZYM and Rapid ID 32A systems, according to the manufacturer’s protocols (bioMérieux Japan Co. Ltd., Tokyo, Japan). Experiments were conducted in duplicate for each microbial strain, LPYR103-Pre^T^ and *Segatella cerevisiae* JCM 30867^T^. In addition, carbon source utilization was evaluated using a batch test [2]. The basal medium was supplemented with 3 g l^−1^ of the carbon source and flushed with N_2_. The growth of strain LPYR103-Pre^T^ on CO_2_ was evaluated through batch testing using a basal medium supplemented with 3 g l^−1^ of pectin and flushed with CO_2_ instead of N_2_. Growth was evaluated by measuring the optical density at 660 nm (OD_660_) and pH after subtracting the OD_660_ of the uninoculated medium. Metabolic SCFAs were evaluated using HPLC, as described previously [2]. Bile sensitivity was determined via the addition of Oxoid bile salts (0.1 or 0.5%, w/v; Thermo Fisher Scientific, Waltham, MA, USA) into pectin agar plates [34]. Cellular fatty acids were converted into methyl esters using the method described by Miller [35]. The methyl esters of cellular fatty acids were analysed using gas chromatography [HP6890 (Hewlett-Packard, Palo Alto, CA, USA) or G-3000 (Hitachi Co. Ltd., Tokyo, Japan)] equipped with an HP Ultra2 column. Cellular fatty acids were identified based on equivalent chain-length comparisons [36,37], according to the protocol of TechnoSuruga Laboratory Co., Ltd. (Shizuoka, Japan) using the MIDI Microbial Identification System (Microbial ID, Inc., Newark, DE, USA) [38].

## Results and discussion

### Phylogenetic position of strain LPYR103-Pre^T^ and its relationship to anaerobic digestion

**Fig. 2.**
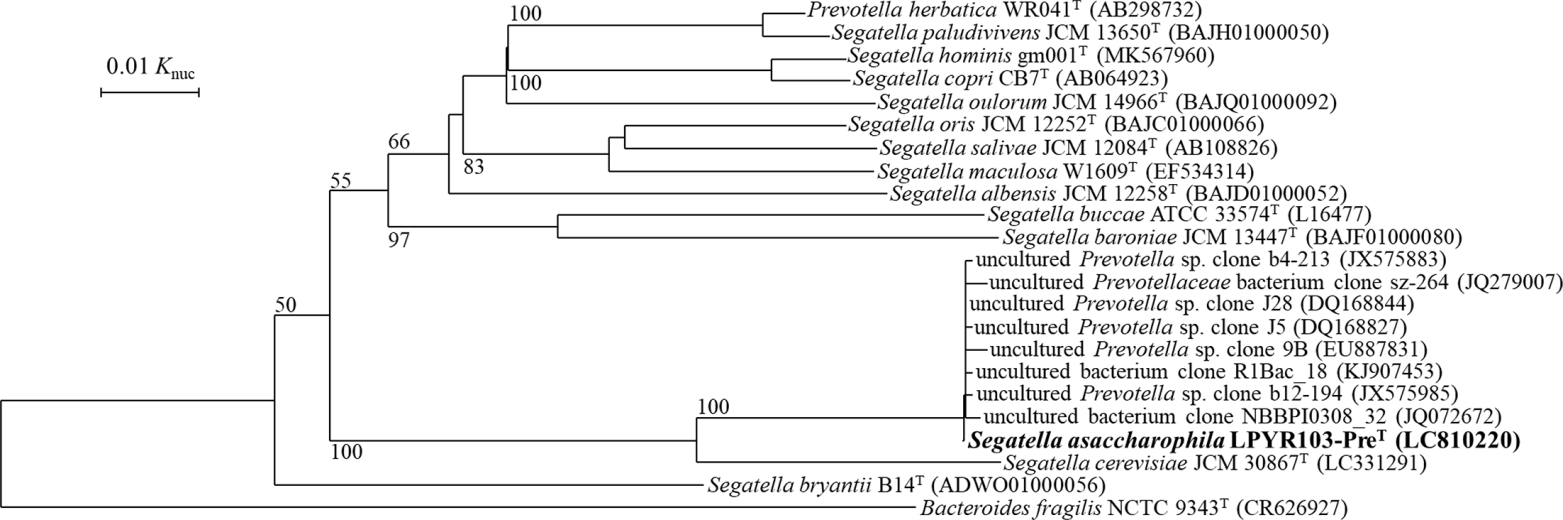
Neighbour-joining tree showing the phylogenetic relationship of *Segatella asaccharophila* LPYR103-Pre^T^, other *Segatella* species, the closest species among its neighbouring genera (*Prevotella herbatica*), and environmental clone sequences based on 16S rRNA gene sequences. The sequence of *Bacteroides fragilis* (Accession number: CR626927) was used as an outgroup. Numbers given at the nodes show bootstrap values >50%, representing the confidence percentage given from 1000 trees. The sequence of *Segatella asaccharophila* LPYR103-Pre^T^, with the accession number LC810220 was subjected to the analysis.

Based on the 16S rRNA gene sequence analysis on the EzBioCloud server, the closest validly described species of the strain LPYR103-Pre^T^ was *Segatella cerevisiae* JCM 30867^T^ [4], with a sequence similarity of 94.4%. The next closest species was *Segatella bryantii* B14^T^ (88.8% similarity) [5]. These 16S rRNA gene sequence similarities were lower than the cut-off value (<98.7 and 97 %) for species delineation [29]. Therefore, strain LPYR103-Pre^T^ formed a distinct branch from *S. cerevisiae* JCM 30867^T^ in the phylogenetic tree (Fig. 2).

Environmental clone sequences related to strain LPYR103-Pre^T^ in the GenBank database were uncultured *Prevotella* sp. clone J5 and clone J28 (DQ168827 and DQ168844; anaerobic sludge), uncultured bacterium clone R1Bac_18 (KJ907453; anaerobic digester), uncultured bacterium clone NBBPI0308_32 (JQ072672; brewery wastewater pilot reactor), uncultured *Prevotella* sp. clone 9B (EU887831; aerobic pre-digester slurry of biogas plant), uncultured *Prevotella* sp. clone b4-213 and clone b12-194 (JX575883 and JX575985; pit mud) and uncultured *Prevotellaceae* bacterium clone sz-264 (JQ279007; groundwater). The sequence similarity of these eight related sequences and strain LPYR103-Pre^T^ were from 99.5 to 99.9%. It is thus possible that strain LPYR103-Pre^T^ is a common inhabitant of anaerobic digesters.

### Strain LPYR103-Pre^T^ is a novel species

The draft genome of strain LPYR103-Pre^T^ consisted of 69 contigs, with a genome length of 3 326 733 bp, an N50 length of 177 631 bp and a genomic DNA G+C content of 44.05% [2]. Comparative assessments against type strains of 12 species belonging to the genus *Segatella*, with genome sizes ranging from 2 668 851 to 4 105 402 bp and genomic DNA G+C contents ranging from 37.56 to 53.04%, revealed that both the genome size and genomic DNA G+C content of strain LPYR103-Pre^T^ were within the normal range of genus *Segatella* (Table 1).

**Table 1.** Comparison of the characteristics of *Segatella asaccharophila* LPYR103-Pre^T^ and other *Segatella* species*

Characteristic	*S. asaccharophila* LPYR103-Pre^T^	*S. cerevisiae* [4]	*S. bryantii* [5,39,41]	*S. albensis* [5,40, 42]	*S. baroniae* [6]	*S. buccae* [7,40, 43]	*S. copri* [8,9, 39, 44]	*S. hominis* [9]	*S. maculosa* [10]	*S. oris* [7,40, 43]	*S. oulorum* [11,42]	*S. paludivivens* [12]	*S. salivae* [13]
Habitat	Anaerobic digestion sludge	Brewery wastewater	Rumen	Rumen	Human oral cavity	Human oral cavity	Human faeces	Human faeces	Human oral cavity	Human oral cavity	Human oral cavity	Plant residue in rice-field soil	Human oral cavity
Growth (optimum range)													
Temperature (°C)	25–37 (34–36)	30, 37‡	37	37	37	37	37	37–42	37	37	37	10–40 (30)	37
pH	5.6–6	5–7	nr	6.7–6.9	nr	nr	6–8	6–9	nr	nr	6.8	4.7–7.6 (5.7–6.7)	nr
Utilization of:													
Glucose	–	+	+	+	+	+	+	+	+	+	+	+	+
Lactose	–	+	+	+	+	+	+	+	+	+	+	+	+
Sucrose	–	+	+	–(+)	+	+	+	+	+	+	+	+	+
Maltose	–	+	+	nr	+	+	+	+	+	+	+	+	+
Salicin	–	+	+	+	+	+(–)	+(–)	–	+	+(–)	–	+	+
Xylose	–	+‡	+(w)	+	–	+	+(–)	+	+	+	–	+	+
Arabinose	–	+	+(w)	+	–	+(–)	+(–)	+	+	+(–)	–	+	+
Cellobiose	–	+	+	+	+	+	+	+	+	+(w)	–	+	+
Mannose	–	+	+	–(+)	+	+	–(+)	+	+	+	+	+	+
Raffinose	–	+	+	–(+)	+	+	+	+	+	+	+	nr	+
Rhamnose	–	+‡	+(w)	–	–	+(–)	+(–)	+	+	–(+, w)	–	+	–
Enzymatic activities:													
API ZYM system													
*β*-Glucuronidase	+	–	–	–	nr	nr	nr	–	nr	nr	–	nr	–
*α*-Fucosidase	–	+‡	nr	–	nr	nr	–	+	nr	nr	–	nr	w
Rapid ID 32A strips													
*β*-Glucuronidase	–	–	–	–	+	–	–	–	+	–	–	–	nr
*α*-Fucosidase	–	–	–	–	+	–	–	+	–	+	–	–	nr
SCFAs produced†	S, A	S, A	S, A, L (F, P, iB, B, iV)	S, A (F, P, iB, iV)	S, A (iB, iV)	S, A (iB, iV)	S, A (P, iB, B, iV)	S, A	S, A	S, A (iB, iV)	S, A	S, A (F)	S, A (iV)
Major cellular fatty acids	anteiso-C_15 : 0_, C_16 : 0_ 3-OH, C_16 :0_	anteiso-C_15 : 0_, C_18 : 1_ ω9c, C_16 : 0_ 3-OH	anteiso-C_15 : 0_, C_15 : 0_, iso-C_15 : 0_	anteiso-C_15 : 0_, C_15 : 0_	anteiso-C_15 : 0_, iso-C_15 : 0_, C_16 : 0_, anteiso-C_17 : 0_	anteiso-C_15 : 0_	anteiso-C_15 : 0_, C_18 : 1_ ω9c, C_16 : 0_	anteiso-C_15 : 0_, iso-C_15 : 0_, C_15 :0_	anteiso-C_15 : 0_, C_16 : 0_, iso-C_15 : 0_, iso-C_17 : 0_	anteiso-C_15 : 0_	anteiso-C_15 : 0_, iso-C_15 : 0_, iso-C_17 :0_	anteiso-C_15 : 0_, iso-C_15 :0_, C_15 : 0_, iso-C_17 : 0_ 3-OH	anteiso-C_15 : 0_, iso-C_17 : 0_ 3-OH, C_18 : 1_ ω9c
Genome size (bp)§	3 326 733	3 288 379	3 558 912	2 668 851	3 128 717	3 284 105	3 512 473	4 105 402	3 314 235	3 168 212	2 840 943	3 449 113	3 274 581
Genomic DNA G+C content (%)§	44.05	43.73	38.92	41.23	53.04	51.02	44.85	42.94	47.39	43.98	46.76	37.36	41.37

*Symbols: +, positive; −, negative; w, weak; (+), some strains are positive; (−), some strains are negative; (w), some strains are weak; (+, w), some strains are positive or weak; nr, not reported. Some information about *Segatella* species was cited from Bac*Dive* (*S. bryantii* with Bac*Dive*-ID:12536; *S. albensis* with Bac*Dive*-ID:12535; *S. buccae* with Bac*Dive*-ID:12526; *S. copri* with Bac*Dive*-ID:12547; *S. oris* with Bac*Dive*-ID:12550; *S. oulorum* with Bac*Dive*-ID:143565; *S. paludivivens* with Bac*Dive*-ID:12558) [45].

†S: succinate; A: acetate; L: lactate; F: formate; P: propionate; iB: isobutyrate; B: butyrate; iV: isovalerate. Fatty acids in parentheses were only found in low or trace amounts.

‡Data from our study. Originally described negative for utilization of xylose and rhamnose and activity of α-fucosidase [4].

§Data from the genomes of type strains of 12 species belonging to the genus *Segatella*. Genome sequence data used in this study were *S. albensis* DSM 11370T (AUFP00000000.1), *S. baroniae* DSM 16972T (AUFQ00000000.1), *S. bryantii* B14T (FOEM00000000.1), *S. buccae* ATCC 33574T (AEPD00000000.1), *S. cerevisiae* DSM 100619T (JAMXLY000000000.1), *S. copri* DSM 18205T (ACBX00000000.2), *S. hominis* BCRC 81118T (SGVY00000000.1), *S. maculosa* DSM 19339T (ARNR00000000.1), *S. oris* NCTC 13071T (LR134384.1), *S. oulorum* ATCC 43324T (FUXK00000000.1), *S. paludivivens* JCM 13650T (BAJH00000000.1) and *S. salivae* DSM 15606T (AEQO00000000.1).

**Fig. 3.**
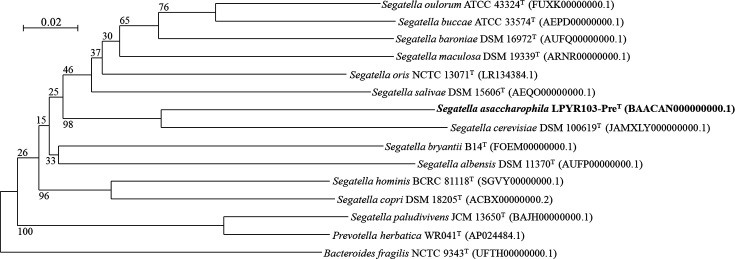
Whole-genome sequence-based Genome Blast Distance Phylogeny (GBDP) tree showing the phylogenetic relationship of *Segatella asaccharophila* LPYR103-Pre^T^, other *Segatella* species and the closest species among its neighbouring genera (*Prevotella herbatica*). The sequence of *Bacteroides fragilis* (Accession number: UFTH00000000.1) was used as an outgroup. The branch lengths are scaled in terms of GBDP distance formula *d_5_*. The bar indicates a GBDP distance of 0.02 upon the application of formula *d_5_*. Numbers given at the nodes show GBDP pseudo-bootstrap support values from 100 replications, with an average branch support of 53.9 %. The sequence of *Segatella asaccharophila* LPYR103-Pre^T^ with the accession number BAACAN000000000.1 was subjected to the analysis. The tree was rooted at the outgroup strain.

The ANI and dDDH values between strain LPYR103-Pre^T^ and other *Segatella* species (*S. cerevisiae* and *S. bryantii*) were 77.9 [2] and 68.8% for ANI and 23.4 and 19.1% for dDDH, respectively (Table S1, available in the online version of this article). These overall genome-related indices for strain LPYR103-Pre^T^ and other *Segatella* type strains were lower than the species-level cut-off values for both ANI (<95–96 %) and dDDH (<70 %) [29]. To further support the observed phylogenetic position of strain LPYR103-Pre^T^, we complemented the 16S rRNA gene sequence-based phylogenetic tree (Fig. 2) with a whole-genome sequence-based phylogenomic tree (Fig. 3). The results showed good agreement with the 16S rRNA gene sequence phylogeny, indicating the positioning of strain LPYR103-Pre^T^ as a novel species.

### Strain LPYR103-Pre^T^ is asaccharolytic and pectinophilic

**Fig. 4.**
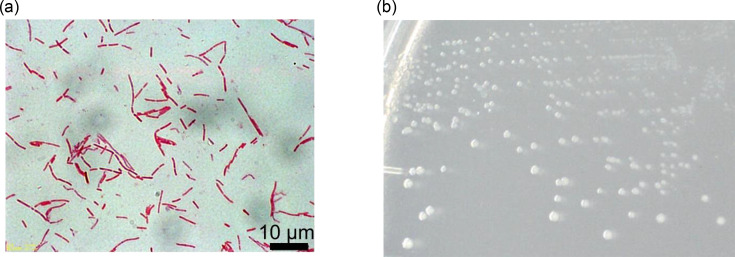
Cell morphology after Gram staining and colony morphology on the LYPP agar plate of *Segatella asaccharophila* LPYR103-Pre^T^. (**a**) Cell morphology. (**b**) Colony morphology.

**Fig. 5.**
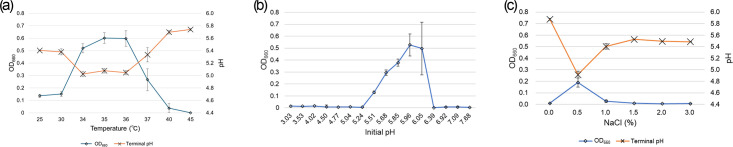
Effect of incubation temperature, initial pH and NaCl concentrations on the growth potential of *Segatella asaccharophila* LPYR103-Pre^T^ (*n*=3). (**a**) Incubation temperature, (**b**) initial pH and (**c**) NaCl concentration. OD_660_: optical density at 660 nm. Error bars indicate standard deviation.

Strain LPYR103-Pre^T^ did not grow in the presence of oxygen and should therefore be considered obligately anaerobic. Strain LPYR103-Pre^T^ is a Gram-stain-negative, non-spore-forming and non-motile bacterium. Cells of strain LPYR103-Pre^T^ grown on the LYPP agar plate were rod-shaped and 0.5×1–7 µm in size (Fig. 4a). Colonies were 0.5–1 mm in diameter, milky white, circular, entire, convex, smooth and opaque on the LYPP agar plate under anaerobic conditions (Fig. 4b). The temperature range for growth was 25–37 °C, with optimum growth at 34–36 °C (Fig. 5a). This suggests that strain LPYR103-Pre^T^ is a mesophilic bacterium, consistent with other *Segatella* species (Table 1). The pH range for growth was between 5.51 and 6.05, with optimum growth at pH 5.96 (Fig. 5b), which is within the normal pH range for growth of the genus *Segatella* (pH 4.7–9; Table 1). Strain LPYR103-Pre^T^ exhibited no growth in the absence of sodium ions; however, it exhibited good growth in the presence of 0.5% NaCl or sodium lactate (approximately 0.1 M as sodium ions; Fig. 5c).

Strain LPYR103-Pre^T^ could be differentiated from its nearest phylogenetic neighbour, *S. cerevisiae* JCM 30867^T^, based on several features: (1) its inability to ferment glucose, lactose, sucrose, maltose, salicin, xylose, arabinose, cellobiose, mannose, raffinose and rhamnose in API 20A strips; (2) its activity for *β*-glucuronidase in the API ZYM system and (3) its inactivity for α-fucosidase in the API ZYM system (Fig. S1a–c, Tables 1 and S2–S4).

**Fig. 6.**
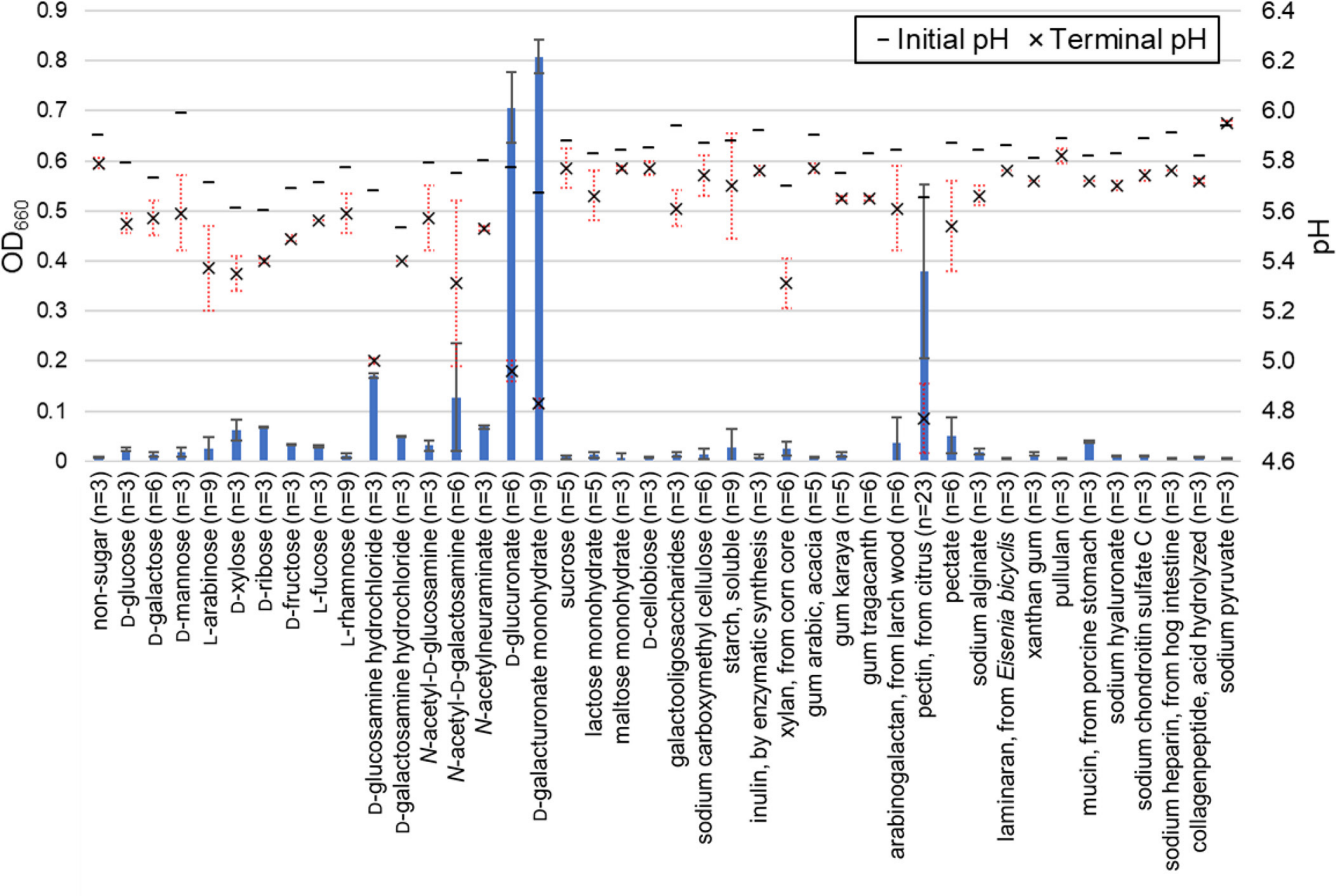
Effect of carbon sources on the growth potential of *Segatella asaccharophila* LPYR103-Pre^T^. OD_660_: optical density at 660 nm. Error bars indicate standard deviation.

Batch tests revealed that the addition of pectin (from citrus), d-galacturonate (monohydrate) or d-glucuronate resulted in the growth of strain LPYR103-Pre^T^ [2]. This growth was slightly stimulated by the addition of d-glucosamine (hydrochloride) and *N*-acetyl-d-galactosamine (Fig. 6). Strain LPYR103-Pre^T^ exhibited poor growth in the presence of d-glucose, d-galactose, d-mannose, l-arabinose, d-xylose, d-ribose, l-fucose, l-rhamnose and pectate [2]. Moreover, strain LPYR103-Pre^T^ exhibited poor growth, following the addition of d-fructose, d-galactosamine (hydrochloride), *N*-acetyl-d-glucosamine, *N*-acetylneuraminate, sucrose, lactose (monohydrate), maltose (monohydrate), d-cellobiose, galactooligosaccharides, sodium carboxymethyl cellulose, starch (soluble), inulin (by enzymatic synthesis), xylan (from corn core), gum arabic (acacia), gum karaya, gum tragacanth, arabinogalactan (from larch wood), sodium alginate, laminaran (from *Eisenia bicyclis*), xanthan gum, pullulan, mucin (from porcine stomach), sodium hyaluronate, sodium chondroitin sulphate C, sodium heparin (from hog intestine), collagen peptide (acid hydrolysed) or sodium pyruvate (Fig. 6). Pectinophiles exhibit specificity in utilizing pectin and a limited range of related compounds as substrates [14]. Hence, these results suggest that strain LPYR103-Pre^T^ can be classified as an anaerobic pectinophile; this strain could be differentiated from other species of *Segatella* based on its preference for a very narrow substrate spectrum for growth, restricted only to pectin and related compounds, such as d-galacturonate and d-glucuronate (Table 1). Strain LPYR103-Pre^T^ exhibited growth in the absence of CO_2_; however, the growth of this strain was enhanced in the presence of CO_2_. Strain LPYR103-Pre^T^ did not grow in the presence of 0.1% (w/v) bile salts.

The major SCFAs produced by strain LPYR103-Pre^T^ were succinate and acetate, consistent with other *Segatella* species (Table 1). While pectinophiles in other genera are known to produce mainly acetate and formate, strain LPYR103-Pre^T^ is unique in producing succinate but not formate (Table 2). Cellular fatty acids analysis revealed that the most abundant cellular fatty acid was anteiso-C_15 : 0_ (35.8%), which is consistent with other *Segatella* species (Table 1). In addition, strain LPYR103-Pre^T^ contained C_16 : 0_ 3-OH (8.2%), C_16 : 0_ (7.0%), iso-C_15 : 0_ (6.5%), iso-C_16 : 0_ (6.4%), anteiso-C_17 : 0_ 3-OH (5.5%) and summed feature 11 (iso-C_17 : 0_ 3-OH and/or C_18 : 2_ DMA; 9.2%) as the predominant fatty acids (representing >5 % of the total). Strain LPYR103-Pre^T^ could be differentiated from *S. cerevisiae* based on C_18 : 1_ ω9c (Table 1).

**Table 2.** Comparison of the characteristics of *Segatella asaccharophila* LPYR103-Pre^T^ and pectinophiles known in other genera*

Characteristic	*S. asaccharophila* LPYR103-Pre^T^	*Lachnospira pectinoschiza* [14]	*Bacteroides pectinophilus* [15,16]	*Bacteroides galacturonicus* [15,16]	*Treponema pectinovorum* [17]	*Eubacterium* sp. strain P-1 [18]	*Monoglobus pectinilytic* [19]	*Natranaerovirga pectinivora* [20,21]	*Natranaerovirga hydrolytica* [20,21]
Oxygen tolerance	Obligately anaerobic	Obligately anaerobic	Obligately anaerobic	Obligately anaerobic	Obligately anaerobic	Obligately anaerobic	Obligately anaerobic	Obligately anaerobic	Obligately anaerobic
Habitat	Anaerobic digestion sludge	Pig intestine	Human intestine	Human intestine	Human oral cavity	Fish intestine	Human faeces	Hypersaline soda lakes	Hypersaline soda lakes
Growth									
Temperature (°C)	25–37	30–45	35–40	35–40	37	15–37	25–40	43	45
pH	5.6–6	nr	7.1	7.1	7	5.5–8	6–8.5	7.2, 8–10.5	7.2, 8.2–10.6
Utilization of:									
Pectin	+	+	+	+	+	+	+	+	+
Galacturonate	+	–	–	+	+	+	+	+	+
Glucuronate	+	–	–	+	+	nr	nr	–	+
Glucose	–	–	–	–	–	–	–	+	–
Galactose	–	–	–	–	–	–	–	–	–
Mannose	–	–	–	–	–	–	–	–	–
Arabinose	–	–	–	–	–	nr	+	–	–
Xylose	–	–	–	–	–	–	+	–	–
Ribose	–	–	–	–	–	nr	nr	+	–
Rhamnose	–	–	–	–	–	nr	–	–	–
Fructose	–	+	+	–	–	–	+	–	+
Sucrose	–	–	–	–	–	–	–	–	–
Lactose	–	+	–	–	–	–	–	+	+
Maltose	–	–	–	–	–	+	–	+	–
Cellobiose	–	+	–	–	–	+	–	–	–
CMC	–	–	–	–	nr	nr	nr	–	–
Starch	–	–	–	–	–	+	–	–	–
Inulin	–	–	–	–	–	nr	–	nr	nr
Xylan	–	–	–	–	nr	–	–	–	–
Arabinogalactan	–	–	–	–	nr	nr	nr	nr	nr
Alginate	–	nr	–	–	nr	–	nr	–	–
Pectate	–	+	+	+	+	nr	nr	+	+
SCFAs produced†	S, A	A, F	A, F (L)	A, F (L)	A, F (L)	A, F	A, F, L	A, F	A, F

*Symbols: +, positive; −, negative; nr, not reported.

†S: succinate; A: acetate; F: formate; L: lactate. Fatty acids in parentheses were only found in low or trace amounts.

In summary, based on the phylogenetic, genomic, physiological and chemotaxonomic characteristics, we conclude that strain LPYR103-Pre^T^ represents a novel species within the genus *Segatella*, for which the name *Segatella asaccharophila* sp. nov. is proposed, with strain LPYR103-Pre^T^ (= NRIC 0997^T^ = JCM 37351^T^=DSM 118531^T^ = KCTC 25923^T^) as the type strain.

### Description of *Segatella asaccharophila* sp. nov.

*Segatella asaccharophila* (a.sac.cha.ro′phi.la. Gr. pref. *a-*, not; Gr. neut. *saccharon*, sugar; Gr. masc. adj. *philos*, loving; N.L. fem. adj. *asaccharophila*, not sugar-loving).

Cells are obligately anaerobic, Gram-stain-negative, non-spore-forming and non-motile rods (0.5×1–7 µm). After 7 days of incubation on the LYPP agar plate, the colonies are 0.5–1 mm in diameter, milky white, circular, entire, convex, smooth and opaque. Growth occurs between 25 and 37°C and pH 5.6–6.0, with optimum growth at 34–36°C and pH 5.96. Good growth in the presence of 0.5% NaCl. Utilizes pectin, d-galacturonate and d-glucuronate as substrate. d-Glucosamine and *N*-acetyl-d-galactosamine can also be utilized. Does not utilize d-glucose, d-galactose, d-mannose, l-arabinose, d-xylose, d-ribose, d-fructose, l-fucose, l-rhamnose, d-galactosamine, *N*-acetyl-d-glucosamine, *N*-acetylneuraminate, sucrose, lactose, maltose, d-cellobiose, galactooligosaccharides, carboxymethyl cellulose, starch, inulin, xylan, gum arabic, gum karaya, gum tragacanth, arabinogalactan, pectate, alginate, laminaran, xanthan gum, pullulan, mucin, hyaluronate, chondroitin sulphate C, heparin, collagen peptide, pyruvate, d-mannitol, salicin, glycerol, d-melezitose, d-raffinose, d-sorbitol or d-trehalose. Growth is enhanced in the presence of CO_2_ and inhibited in the presence of bile salts. The major SCFAs produced by strain LPYR103-Pre^T^ are succinate and acetate. The most abundant cellular fatty acid is anteiso-C_15 : 0_.

In API 20A strips, the strain hydrolyses aesculin, but not gelatin. Tests are negative for indole formation, urease and catalase activity. In the API ZYM system, positive reactions are obtained for alkaline phosphatase, leucine arylamidase, acid phosphatase, naphthol-AS-BI-phosphohydrolase, α-galactosidase, β-galactosidase, β-glucuronidase, α-glucosidase, β-glucosidase and *N*-acetyl-β-glucosaminidase, but negative for esterase, esterase lipase, lipase, valine arylamidase, cystine arylamidase, trypsin, α-chymotrypsin, α-mannosidase and α-fucosidase.

In Rapid ID 32A strips, positive reactions are obtained for *α*-galactosidase, *β*-galactosidase, *α*-glucosidase, *β*-glucosidase, *α*-arabinosidase, *N*-acetyl-*β*-glucosaminidase, alkaline phosphatase, leucyl glycine arylamidase and alanine arylamidase. Negative reactions are obtained for urease, arginine dihydrolase, *β*-galactosidase-6-phosphate, *β*-glucuronidase, glutamic acid decarboxylase, α-fucosidase, nitrate reduction, indole production, arginine arylamidase, proline arylamidase, phenylalanine arylamidase, leucine arylamidase, pyroglutamic acid arylamidase, tyrosine arylamidase, glycine arylamidase, histidine arylamidase, glutamyl glutamic acid arylamidase and serine arylamidase.

The type strain is LPYR103-Pre^T^ (= NRIC 0997^T^ = JCM 37351^T^=DSM 118531^T^ = KCTC 25923^T^), isolated from anaerobic digestion sludge obtained from a two-phase methane fermentation system equipped with solubilization treatment at Tokyo University of Agriculture, Japan. The genome size DNA and G+C content of the type strain are 3 326 733 bp and 44.05%, respectively. The GenBank accession numbers for the 16S rRNA gene and genome sequences of strain LPYR103-Pre^T^ are LC810220 and BAACAN000000000.1 (BioProject accession No. PRJDB17761), respectively.

## Supplementary material

10.1099/ijsem.0.006606 Uncited Supplementary Material 1.

## Abbreviations

ANI: average nucleotide identity
dDDH: digital DNA–DNA hybridization
GBDP: Genome Blast Distance Phylogeny
LYPP: lactate–yeast extract–peptone–pectin
SCFAs: short-chain fatty acids
