# Supplementary material for: Segatella asaccharophila sp. nov., an anaerobic pectinophile isolated from a two-phase methane fermentation system
Source: Int J Syst Evol Microbiol. 2024 Dec 18;74(12):006606. doi: 10.1099/ijsem.0.006606 (PMC12509415; doi:10.1099/ijsem.0.006606)
Supplement: Uncited Supplementary Material 1. [file ijsem-74-06606-s001.pdf]

Segatella asaccharophila sp. nov., an anaerobic pectinophile isolated from a two-phase methane fermentation system

Supplementary information

Supplementary Figures

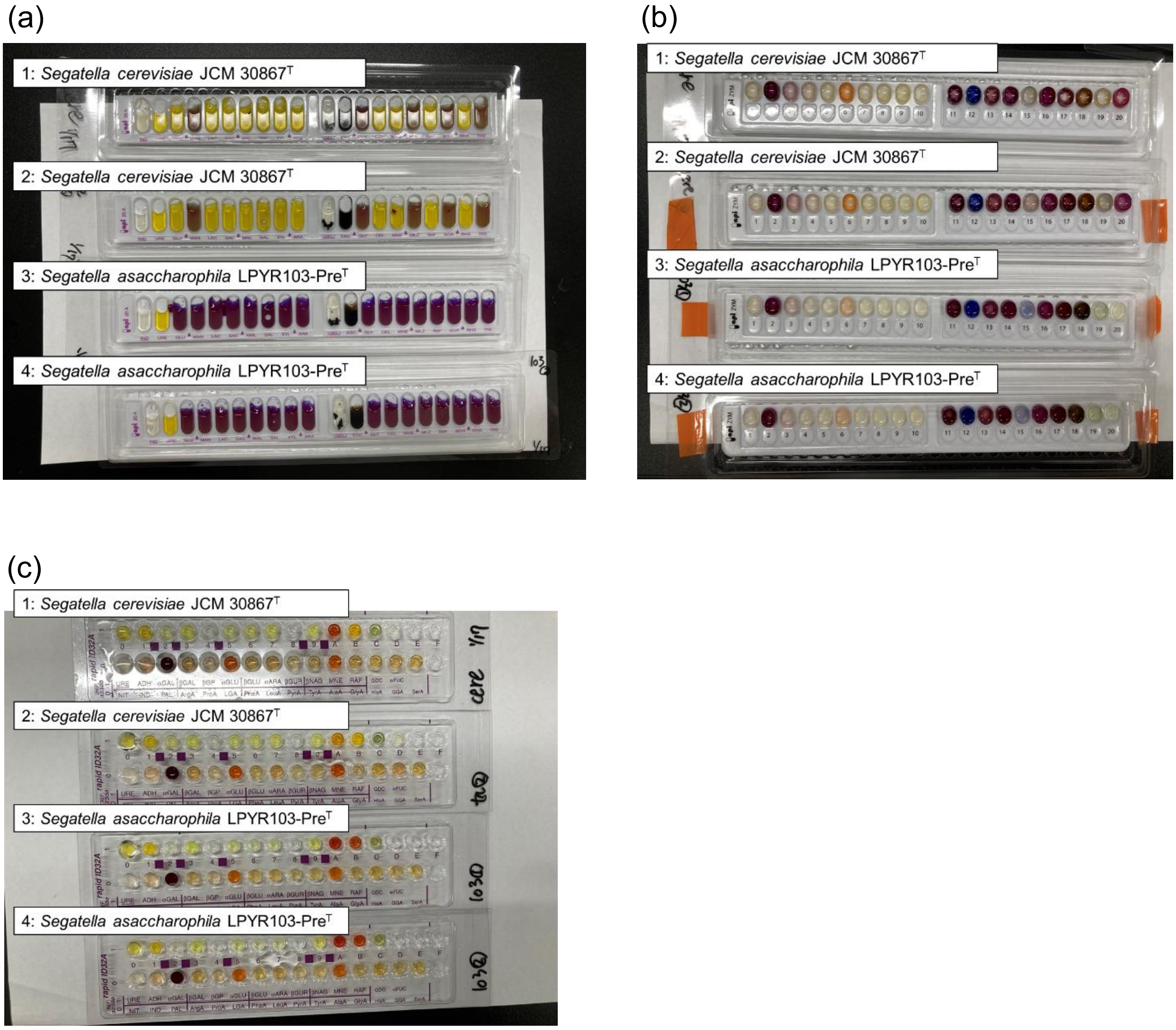

Fig. S1. API 20 A, API ZYM, and rapid ID 32A system profiles of *Segatella cerevisiae* JCM 30867^{T} and *Segatella asaccharophila* LPYR103-Pre^{T}.
(a) API 20 A, (b) API ZYM, (c) rapid ID 32A

Supplementary Tables

Table S1. ANI and dDDH values between the genomes of *Segatella asaccharophila* LPYR103-Pre^{T} and other *Segatella* species.

Taxon name	Strain name	Accession number	ANI (%)	dDDH (%) (Formula 2)
Segatella albensis	DSM 11370^{T}	AUFP00000000.1	69.2	17.4
Segatella baroniae	DSM 16972^{T}	AUFQ00000000.1	68.4	19.5
Segatella bryantii	B14^{T}	FOEM00000000.1	68.8	19.1
Segatella buccae	ATCC 33574^{T}	AEPD00000000.1	69.1	17.9
Segatella cerevisiae	DSM 100619^{T}	JAMXLY000000000.1	77.9	23.4
Segatella copri	DSM 18205^{T}	ACBX00000000.2	69.2	19.4
Segatella hominis	BCRC 81118^{T}	SGVY00000000.1	69.6	19.3
Segatella maculosa	DSM 19339^{T}	ARNR00000000.1	68.1	19.8
Segatella oris	NCTC 13071^{T}	LR134384.1	68.6	21.1
Segatella oulorum	ATCC 43324^{T}	FUXK00000000.1	67.7	20.5
Segatella paludivivens	JCM 13650^{T}	BAJH00000000.1	68.6	18.8
Segatella salivae	DSM 15606^{T}	AEQO00000000.1	68.2	19.1

Table S2. API 20A strips profile of *Segatella cerevisiae* JCM 30867^{T} and *Segatella asaccharophila* LPYR103-Pre^{T}.

	Segatella cerevisiae JCM 30867^{T}		*Segatella asaccharophila* LPYR103-Pre^{T}	
	1	2	3	4
indole production	−	−	−	−
urease	−	−	−	−
**Acidification:**				
D-glucose	+	+	−	−
D-mannitol	−	−	−	−
D-lactose	+	+	−	−
sucurose	+	+	−	−
D-maltose	+	+	−	−
salicin	+	+	−	−
D-xylose	+	+	−	−
L-arabinose	+	+	−	−
**Hydrolysis:**				
gelatin	−	−	−	−
esculin	+	+	+	+
**Acidification:**				
glycerol	−	−	−	−
D-cellobiose	+	+	−	−
D-mannose	+	+	−	−
D-melezitose	−	−	−	−
D-raffinose	+	+	−	−
D-sorbitol	−	−	−	−
L-rhamnose	+	+	−	−
D-trehalose	−	−	−	−
catalase	−	−	−	−

+, Positive; −, negative

Table S3. API ZYM system profile of *Segatella cerevisiae* JCM 30867T and *Segatella asaccharophila* LPYR103-PreT.

	Segatella cerevisiae JCM 30867T		*Segatella asaccharophila* LPYR103-PreT	
	1	2	3	4
alkaline phosphatase	+	+	+	+
esterase	−	−	−	−
esterase lipase	−	−	−	−
lipase	−	−	−	−
leucine arylamidase	+	+	+	+
valine arylamidase	−	−	−	−
cystine arylamidase	−	−	−	−
trypsin	−	−	−	−
α-chymotrypsin	−	−	−	−
acid phosphatase	+	+	+	+
naphthol-AS-BI-phosphohydrolase	+	+	+	+
α-galactosidase	+	+	+	+
β-galactosidase	+	+	+	+
β-glucuronidase	−	−	+	+
α-glucosidase	+	+	+	+
β-glucosidase	+	+	+	+
N-acetyl-β-glucosaminidase	+	+	+	+
α-mannosidase	−	−	−	−
α-fucosidase	+	+	−	−

+, Positive; −, negative

Table S4. rapid ID 32A strips profile of *Segatella cerevisiae* JCM 30867^{T} and *Segatella asaccharophila* LPYR103-Pre^{T}.

	Segatella cerevisiae JCM 30867^{T}		*Segatella asaccharophila* LPYR103-Pre^{T}	
	1	2	3	4
urease	−	−	−	−
arginine dihydrolase	−	−	−	−
α-galactosidase	+	+	+	+
β-galactosidase	+	+	+	+
β-galactosidase-6-phosphate	−	−	−	−
α-glucosidase	+	+	+	+
β-glucosidase	+	+	+	+
α-arabinosidase	+	+	+	+
β-glucuronidase	−	−	−	−
N-acetyl-β-glucosaminidase	+	+	+	+
mannose fermentation	−	+	−	−
raffinose fermentation	+	+	−	−
glutamic acid decarboxylase	−	−	−	−
α-fucosidase	−	−	−	−
nitrate reduction	−	−	−	−
indole production	−	−	−	−
alkaline phosphatase	+	+	+	+
arginine arylamidase	−	−	−	−
proline arylamidase	−	−	−	−
leucyl glycine arylamidase	+	+	+	+
phenylalanine arylamidase	−	−	−	−
leucine arylamidase	−	−	−	−
pyroglutamic acid arylamidase	−	−	−	−
tyrosine arylamidase	−	−	−	−
alanine arylamidase	+	+	+	+
glycine arylamidase	−	−	−	−
histidine arylamidase	−	−	−	−
glutamyl glutamic acid arylamidase	−	−	−	−
serine arylamidase	−	−	−	−

+, Positive; −, negative
